# Identification and characterization of novel serum microRNA candidates from deep sequencing in cervical cancer patients

**DOI:** 10.1038/srep06277

**Published:** 2014-09-03

**Authors:** Li Juan, Hong-li Tong, Pengjun Zhang, Guanghong Guo, Zi Wang, Xinyu Wen, Zhennan Dong, Ya-ping Tian

**Affiliations:** 1 Department of Clinical Biochemistry, State Key Laboratory of Kidney Disease, Chinese PLA General Hospital, Beijing, China, 100853; 2 These authors contributed equally to this work.

## Abstract

Small non-coding microRNAs (miRNAs) are involved in cancer development and progression, and serum profiles of cervical cancer patients may be useful for identifying novel miRNAs. We performed deep sequencing on serum pools of cervical cancer patients and healthy controls with 3 replicates and constructed a small RNA library. We used MIREAP to predict novel miRNAs and identified 2 putative novel miRNAs between serum pools of cervical cancer patients and healthy controls after filtering out pseudo-pre-miRNAs using Triplet-SVM analysis. The 2 putative novel miRNAs were validated by real time PCR and were significantly decreased in cervical cancer patients compared with healthy controls. One novel miRNA had an area under curve (AUC) of 0.921 (95% CI: 0.883, 0.959) with a sensitivity of 85.7% and a specificity of 88.2% when discriminating between cervical cancer patients and healthy controls. Our results suggest that characterizing serum profiles of cervical cancers by Solexa sequencing may be a good method for identifying novel miRNAs and that the validated novel miRNAs described here may be cervical cancer-associated biomarkers.

Cervical cancer is one of the most common cancers in women and creates a huge burden for women's health in the world, especially in developing countries. Because clinical utility of serum biomarkers for cervical cancer diagnosis is limited, there is an urgent need for a minimally invasive, fast and efficient method to diagnose the disease^1^.

MicroRNAs (miRNAs) are a type of small, non-coding RNA that mediate post-transcriptional gene silencing by binding to the 3′ untranslated region of mRNAs^2^. miRNAs are single-stranded RNAs, are approximately 22 nucleotides long and play important regulatory roles in various biological processes, including cellular proliferation, apoptosis, angiogenesis, invasion and migration^3^. Many studies have provided evidence that varieties of miRNAs are involved with the initiation and progression of human malignancies^4^,^5^,^6^. Recent discoveries have showed that serum and plasma contained a large amount of stable miRNAs derived from various tissues or organs, and identification of these miRNAs was reproducible and consistent among individuals, suggesting miRNAs could be exploited as biomarkers for the diagnosis of cancer and other diseases^7^,^8^,^9^,^10^.

Various studies have reported aberrant expression of miRNAs in cervical cancers compared with normal cervixes^11^,^12^. In a comparison of profiles of miRNAs in six human cervical carcinoma cell lines and five normal cervical samples, six miRNAs were identified with significant expression variation between the two groups, and reduced expression of miR-143 and increased expression of miR-21 were further validated^13^. MiR-19a/b was highly expressed in human cervical cancer cells and directly and negatively regulated CUL5 expression, which highlights the importance of miRNA-19a and miRNA-19b and their target genes in tumorigenesis^14^. MiR-34a was expressed at various levels in cervical cancer and inhibited cancer invasiveness by regulating the Notch pathway^15^. The above cited studies all focused on cervical cancer cells or tissues. Very few studies have emphasized profiles of circulating miRNAs in cervical cancer patients. Thus, characterizing serum profiles of miRNAs between cervical cancer patients and healthy controls by trans-genome sequencing may facilitate the identification of more candidate novel miRNAs and possibly provide new serum markers for cervical cancer early warning, diagnosis and prognosis.

In this study, we performed deep sequencing to analyze serum profiles of miRNA between cervical cancer patients and healthy controls to identify and characterize novel miRNAs. We validated the expressions of 2 predicted novel miRNAs that were identified. We expect that the novel and differentially expressed miRNAs identified in this study could provide a basis for further research of the molecular mechanism underlying the development of cervical cancer.

## Results

### Construction of a small RNA library by Solexa sequencing

Solexa sequencing was performed on the sera of 21 cervical cancer patients and 21 healthy controls. The clinical data of all the subjects are shown in supplementary table S1. Deep sequencing yielded 13191837, 17201872 and 11517031 total reads for the cervical cancer C1, C2 and C3 groups, respectively, and 14530924, 9044505 and 12042843 total reads for the H1, H2 and H3 groups, respectively (Table 1). Removing adaptors, low quality tags and contaminants yielded 96.23% (12655313), 85.49% (14664409) and 93.52% (10730467) of the total reads to further analyze for the C1, C2 and C3 groups, respectively, and 96.84% (14039577), 94.36% (8516261) and 94.20% (11321127) clean reads for the H1, H2 and H3 groups, respectively (Table 1). We then summarized the length distribution of these clean reads. Length distribution analysis showed that most reads were in the range of 18 to 24 nt in serum pools of both the cervical cancer groups and the healthy control groups, which is consistent with the common sizes of miRNAs (Supplementary Fig. S1 and S2). Although the length distributions showed differences between cervical cancer groups and healthy controls as well as differences in the three replicates within each group, we observed that miRNAs in the range of 21 nt to 23 nt account for the highest percentage of clean reads.

### Common and specificunique small RNA (sRNAs) sequences in cervical cancer patients and healthy controls

The common and unique tags in the 6 serum pools are summarized in supplementary Figure S3. Analysis showed that there were only 6.59% uniques RNAs in common between C1 and H1, 4.92% between C2 and H2 and 4.87% between C3 and H3. There were large differences of unique sRNAs among cervical cancer and healthy control samples, but the percentage of common total sRNAs between cervical cancer and healthy control group was high. Analysis showed that 97.16% total sRNAs were in common between C1 and H1, 95.76% between C2 and H2 and 96.78% between C3 and H3 (supplementary Figure S4).

### Unannotated small RNA sequences

All Solexa reads were mapped to the genome using SOAP. Approximately 58.25%, 44.64% and 59.23% of the total reads were matched to the genome in the cervical cancer C1, C2 and C3 groups, respectively, whereas 53.61%, 50.36% and 54.07% of the total reads in the H1, H2 and H3 group were matched with the genome. After alignment to the Genbank database and Rfam, small RNAs were classified into different categories (Table 1). Then, we removed known miRNAs, tRNAs, rRNAs, snoRNAs, snRNAs and repeat associated RNAs. The remaining sequences which did not match any database were considered unannotated sequences. There were 9040059 (71.43%) unannotated small RNAs in C1, 7881033 (53.74%) in C2 and 81129 (39.97%) in C3. There were 6380477 (45.45%) unannotated small RNAs in H1, 4183759 (49.13%) in H2 and 5120372 (45.23%) in H3.

### Repeat analysis among 3 cervical cancer groups and 3 healthy control groups

**Figure 1:**
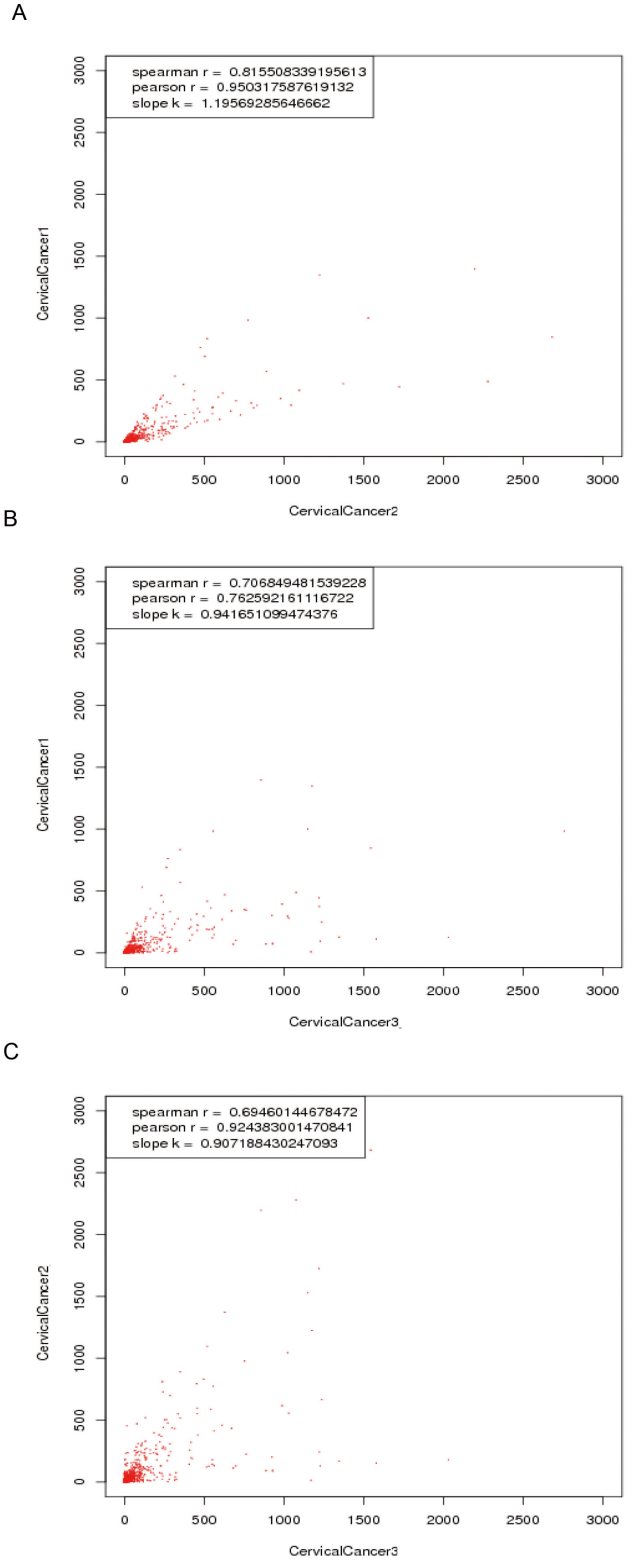
Repeat analysis of the three replicates of serum pools of the cervical cancer group by Solexa sequencing. (A) Correlation analysis between serum pools of cervical cancer group 1(C1) and group 2 (C2). (B) Correlation analysis between serum pools of cervical cancer group 1(C1) and group3 (C3). (C) Correlation analysis between serum pools of cervical cancer group 2 (C2) and group 3 (C3).

Although the three serum pools of cervical cancer have balanced tumor stages, the expression levels measured among the 3 replicates by Solexa sequencing might be somewhat inconsistent for certain miRNAs. Using correlation analysis between each pair of groups, we found that the three groups had good correlation. As shown in Figure 1, the Pearson correlation coefficient between cervical cancer groups 1 and group 2 was 0.95; the Pearson correlation coefficient between cervical cancer groups 1 and 3 was 0.76; and the Pearson correlation coefficient between cervical cancer groups 2 and 3 was 0.92. Correlation analysis between each pair of groups in healthy controls resulted in higher Pearson correlation coefficients (all R > 0.98) (Supplementary Fig. S5).

### Identification of novel miRNAs

We obtained 199 novel miRNAs candidates in total in the cervical cancer groups and 202 novel miRNAs candidates in the healthy control groups which were predicted by MIREAP. There were 17 common novel miRNAs candidates between cervical cancer groups and healthy control groups, and one of these sequences had been registered in miRBase 20.0. Among these16 novel miRNAs common between the cervical cancer and healthy control groups, we selected only novel miRNAs which could be detected twice in the three repeats of each group as putative novel miRNAs, which left 6 novel miRNAs found repeatedly in both groups. Next, we filtered out pseudo-pre-miRNAs using Triplet-SVM methods, leaving only 2 novel miRNAs to be validated. These remaining 2 putative miRNAs were selected for validation by qRT-PCR. The sequences, MFE and length of each predicted novel miRNA are listed in Table 2. To further confirm the existence of miRNA among our sequencing results, we predicted the secondary structure of these precursors of predicted miRNA candidates (Supplementary Fig. S6).

### Real-time PCR validation of novel miRNAs from cervical cancer patients and healthy controls

**Figure 2:**
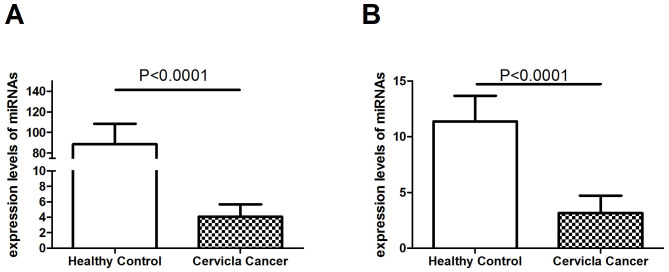
Expression levels of the 2 novel miRNAs (A, PmiR-1; B, PmiR-2) in cervical cancer patients (N = 112) and healthy controls (N = 85) by RT-PCR validation.

Ten cervical cancer patients and 10 healthy controls were selected for the validation of the 2 putative novel miRNAs. The 2 novel miRNAs were both validated in the two groups. Then, 102 cervical cancer patients and 75 age-matched healthy controls were further used for the validation of the 2 predicted miRNAs. The characteristics of the research subjects in the validation set are shown in Supplementary Table S2. The forward primer of each miRNA is presented in Table 2. We chose U6 snRNA as our internal control. The 2 predicted novel miRNAs were confirmed by qRT-PCR. The expression levels of both miRNAs were normalized to U6 snRNA and presented as fold changes (2^−ΔΔCt^). The Mann-Whitney U test was used to compare expression differences between the cervical cancer group and the healthy control group. As shown in Figure 2, serum expression levels of the 2 novel miRNAs were all significantly reduced in cervical cancer patients compared with healthy controls (all p < 0.0001). Serum expression levels of PmiR-1 and PmiR-2 had expressions downregulated > 2-fold (Supplementary Table S3).

Mann-Whitney U test analysis of the 2 novel miRNAs for all cervical cancer patients analyzed in this study indicated that their expressions were not dependent on tumor stage (stage I versus II and III, P > 0.05) (Supplementary Fig. S7), tumor size (≥4 cm or <4 cm, P > 0.05) (Supplementary Fig. S8), lymph node metastasis (negative or positive, P > 0.05) (Supplementary Fig. S9), or tumor grade (G3 versus G1 and G2, P > 0.05) (Supplementary Fig. S10).

### Detection of serum miRNAs from cervical cancer patients and other cancer patients by qRT-PCR analysis

**Figure 3:**
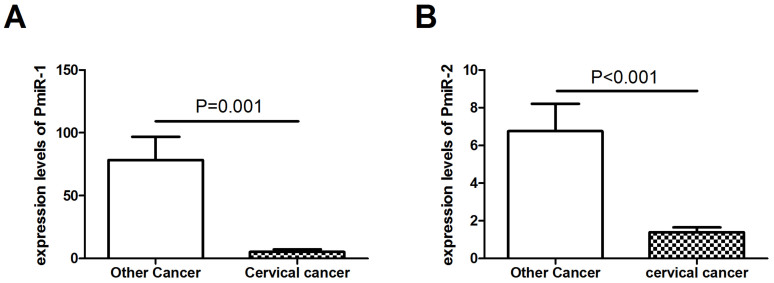
Expression levels of the 2 novel miRNAs (A, PmiR-1; B, PmiR-2) in cervical cancer patients (N = 112) and patients with other cancers (N = 55) by RT-PCR validation.

The two putative novel serum miRNAs were further validated in 55 patients with other cancers including 10 colorectal cancer patients, 16 ovarian cancer patients, 10 breast cancer patients, 7 gastric cancer patients and 12 endometrial cancer patients. The results showed that the expressions of the two miRNAs were significantly decreased between cervical cancer patients and other cancer patients (P < 0.05) (Figure 3).

### Distinction between cervical cancer cases and healthy controls by the 2 miRNA-based biomarkers

**Figure 4:**
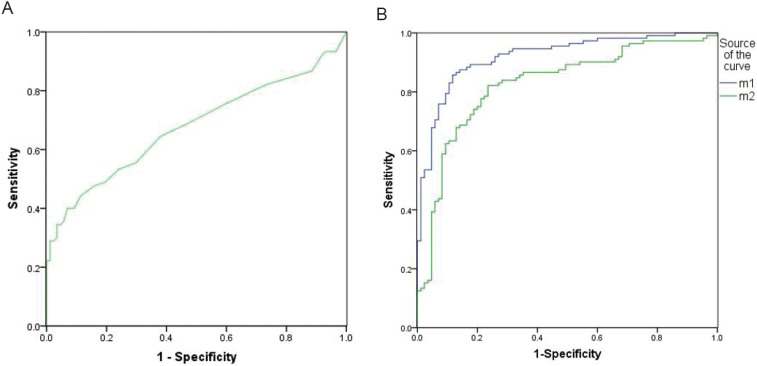
ROC curve analysis of the 2 novel miRNAs and SCC in diagnosing cervical cancer patients (N = 112) from healthy controls (N = 85). (A) SCC (AUC = 0.690, 95% CI:0.605–0.776). (B) m1 represents PmiR-1 (AUC = 0.921, 95% CI:0.883–0.959); m2 represents PmiR-2 (AUC = 0.827, 95% CI: 0.767–0.887).

To test whether the 2 miRNA-based biomarkers could distinguish cervical cancer patients from healthy controls, we performed ROC curve analysis to compare their diagnostic value. The results are shown in Figure 4 and Supplementary Table S4. SCC is the most common biomarker used in clinical practice to help diagnose cervical cancer patients. Therefore, we used ROC analysis to compare SCC and the 2 novel miRNAs. The AUC for SCC distinguishing the cervical cancer group from the healthy controls was 0.690 (95%CI: 0.605–0.776), whereas the AUC values for the 2 novel miRNAs were 0.921(95%CI: 0.883–0.959) and 0.827(95%CI: 0.767–0.887), respectively. The novel miRNA PmiR-2 had the largest AUC at 0.921 when the cut-off value was set at 4.24, the sensitivity was 85.7% and the specificity was 88.2%, suggesting that PmiR-2 has better diagnostic value than SCC.

### Construction of miRNA-gene-network

**Figure 5:**
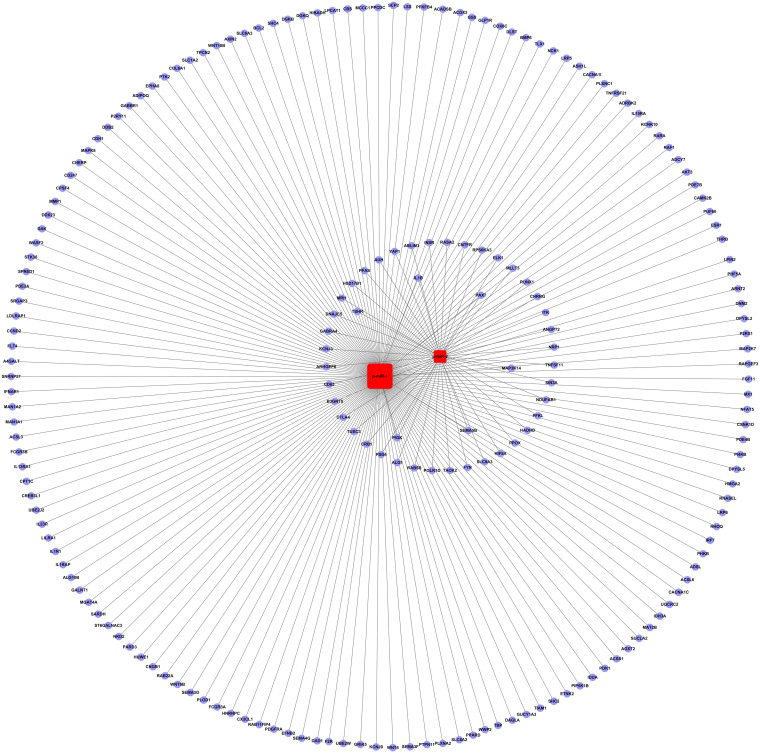
miRNA-gene-network. Circular nodes represent genes, and square nodes represent miRNAs. The top six key genes in the human network were IL-1β, MAP3K14, PAX 7, PIGK, SEMA5B and TSHR.

**Figure 6:**
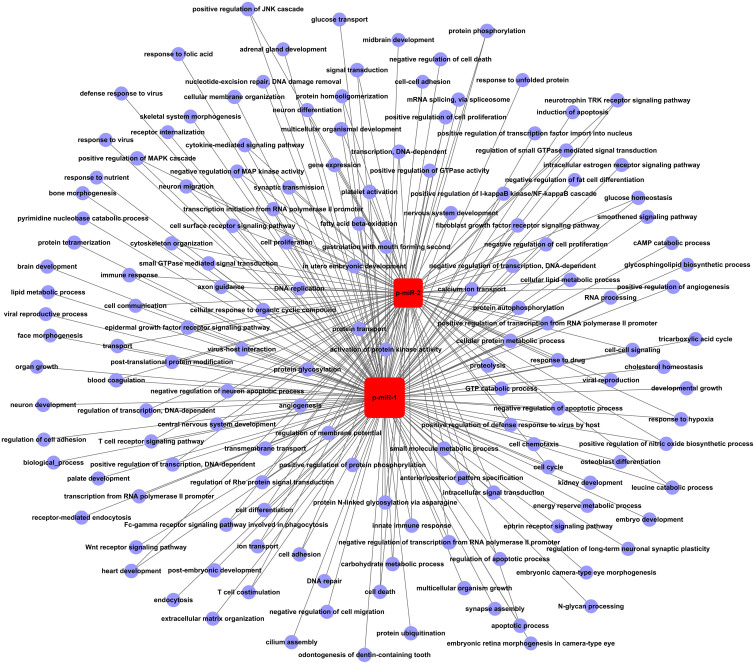
miRNA-GO-network. The miRNA-GO-network was generated according to the relationship of significant functions and miRNAs. Circular nodes represent GOs, and square nodes represent miRNAs. Their relationships are represented by lines.

To further understand the biological functions of the two miRNAs, their target genes were predicted by Targetscan and miRanda. The results indicated 1341 target genes for p-miR-1 and 379 target genes for p-miR-2. We performed GO and pathway enrichment analyses and selected intersected genes. According to the interactions between miRNAs and the intersected genes, we built a miRNA-gene-network which illustrates the key regulatory functions of the identified miRNAs and their target genes (Figure 5). The top six target genes were: IL-1β (interleukin 1 beta), MAP3K14 (mitogen-activated protein kinase kinase kinase 14), PAX 7 (paired box 7), PIGK (phosphatidylinositol glycan anchor biosynthesis class K), SEMA5B (semaphorin 5B) and TSHR (thyroid stimulating hormone receptor). We constructed a miRNA-GO-network to understand the key biological functions of the two miRNAs (Figure 6). Our analysis showed that the two miRNAs may play roles in processes including apoptosis, cell proliferation, angiogenesis, virus-host interaction and innate immune response.

## Discussion

Although cervical cancer is a preventable disease, it is still a common cancer for women worldwide. HPV infection is the principle cause of cervical cancer, but HPV infection is not sufficient to cause cervical cancer^16^. Accumulating evidence indicates that various miRNAs may play critical roles in tumorigenesis^5^,^17^. Comprehensive analysis of miRNA expression between cervical cancer patients and healthy controls will help us to better understand the roles of miRNAs in the development of the disease as well as to find novel biomarkers for cervical cancer diagnosis and prognosis for individualized therapy. Various studies have shown that miRNAs are aberrantly expressed or mutated in several cervical cancer cell lines and tissues^1^,^13^,^16^,^18^. Considering that the collection of tissue samples is an invasive procedure and that surgical sections are always obtained after the initial clinical classification, the use of tissue miRNAs as a cancer biomarker is greatly limited in cancer diagnosis^19^. In recent years, researchers have proposed that serum miRNAs could potentially serve as novel of biomarkers for the detection of various cancers and other diseases^7^,^8^,^20^. RNA sequencing (RNA-Seq) technologies supported by the innovative massively parallel platforms have emerged as powerful tools for the detection of differential gene expressions between samples and for the identification of novel miRNAs in the small RNA transcriptome^21^,^22^,^23^. Thus, serum profiles of miRNAs obtained by high-throughput sequencing may provide a way to find novel miRNAs as biomarkers for cervical cancer diagnosis.

In the present study, we sequenced 6 pooled serum miRNAs of cervical cancer patients and healthy Chinese subjects using a Solexa high-throughput sequencing system with three replicate samples per group. Analysis showed a high correlation between each pair of replicates within cervical cancer patients and within the healthy control group. Length distribution analysis showed that miRNAs with 21 nt to 23 nt accounted for the highest percentage of miRNAs found both in cervical cancer group and healthy control group, consistent with the common size of miRNAs. Some differences in the distribution of miRNAs were observed between the cervical cancer groups and healthy control groups, as well as among the three replicates within each group. We envision a number of possible explanations for the differences among replicates within cervical cancer group or healthy control group^24^. First, the inconsistent distribution among replicates may result from the random sampling of sequencing. Second, natural variations among biological replicates may affect the results. Third, for human studies, sequence polymorphisms among biological replicates may also result in different reads among biological replicates^25^. Therefore, when some reads contained sequence polymorphisms compared with the reference sequences, they were likely to be discarded during mapping^26^. Fourth, some technical factors specific to the RNA-seq procedures, such as the preparation effect of sequencing, uneven sequencing depths^27^ and flow cell and lane effects^28^, may result in the differences we observed. In addition to the above reasons, the variation of length distribution of miRNAs between the cervical cancer group and the healthy control group may be largely affected by specific miRNAs involved in cervical cancer.

In this study, there was a large percentage of unique sRNAs among cervical cancer and healthy control samples by Solexa sequencing. This result may be explained by the dynamic changes of miRNAs in different diseases and at different disease development stages^29^. The results of hundreds of miRNA profiling studies showed that tumors could exhibit altered miRNA expression patterns compared with normal tissue or serum/plasma for tumorigenesis. Furthermore, more than 95% of cervical cancers are caused by persistent HPV infection. There may be large number of microbial, non-human sRNAs in the serum of cervical cancer patients. In addition, because HPV infection appears to be necessary for cervical cancer development, expression of the miRNAs associated with host immunity may be altered greatly during the development of cervical cancer. After length distribution, the common and specific unique sRNAs and total sRNAs were summarized among the cervical cancer groups and healthy control groups. Therefore, analyzing miRNA profiles of cervical cancer patients may provide useful information for cervical cancer diagnosis and prognosis.

In this study, we found 17 common novel miRNAs candidates between the cervical cancer group and the healthy control group predicted by MIREAP. We narrowed our focus to miRNAs which could be detected twice in the 3 replicates in each group. After removing miRNAs which were potential pseudo-pre-miRNAs or had been registered in miRBase, we obtained 2 candidate novel miRNAs for further validation. We also predicted the secondary structure of the precursors of these two putative novel miRNAs. This analysis showed that both putative miRNAs should have the typical hairpin shape. Many studies have identified novel miRNAs by Solexa sequencing, although the methods of computational analysis for novel miRNA prediction may be different from the ones used here^30^,^31^,^32^. Candidate novel miRNAs obtained from deep sequencing must be qualified as “real” hits by further validation. In this study, the 2 novel miRNAs were well validated in the serum of 112 cervical cancer patients and 85 age-matched healthy controls by RT-PCR. Serum expression comparison revealed that the 2 novel miRNAs were significantly down-regulated in the cervical cancer group compared with the healthy control group. The expressions of the 2 novel miRNAs were not correlated with tumor stage, lymph node metastasis or pathological differentiation (all P > 0.05), which indicates that, although the aberrant expression of the 2 miRNAs may be unrelated to the advancement of cancer, the expression of these miRNAs may be related to the occurrence of cervical cancer. These results were consistent with a study by Qunxian Rao et al., which found miRNA expression was independent of lymph node involvement, vascular invasion, and pathological differentiation^18^. Analysis of an miRNA-gene-network revealed that IL-1β, MAP3K14, PAX 7, PIGK, SEMA5B and TSHR were key target genes for the 2 novel miRNAs. GO analysis showed that these miRNAs may be involved in apoptosis, cell proliferation, angiogenesis, virus-host interaction or innate immune response.

We further explored the clinical utility of the 2 novel miRNAs in distinguishing cervical cancer patients from healthy controls. ROC curve analysis show that one of the identified miRNAs had good AUC (>0.90) with high sensitivity and specificity. Although the biological function of the 2 novel miRNAs in cervical cancer is unknown, their differential expression between cervical cancers and healthy controls enables clinical utility in separating the two groups, suggested that the 2 novel miRNAs may serve as cervical cancer-associated biomarkers for diagnosis.

Several findings of this study are noteworthy. First, few investigators to date have sequenced serum profiles of cervical cancer patients and identified novel miRNAs by deep sequencing. Our study is the first analysis of serum miRNAs in cervical cancer at different stages by Solexa sequencing. Furthermore, we made serum profiles of the cervical cancer group and of the healthy control group with 3 replicates in each and analyzed their correlation. Biological replicates and procedural replicates were vital for robust statistical inference of differential expression. Averaging across replicates can increase the precision of gene expression measurements. José A. Robles and colleagues have showed that greater power is gained through the use of biological replicates compared to library (technical) replicates and sequencing depth for RNA sequencing^22^. However, to date few studies have incorporated extensive biological replication in their experiments when using trans-genome sequencing to compare profiles of miRNAs. Therefore, the application of replicates for Solexa sequencing is one aspect of the highlights in our study.

There were also some limitations to our present study. First, we only made serum profiles of cervical cancer and healthy controls. Supplementing these data with miRNAs profiles of pre-cancerous benign lesions (Cervical Intraepithelial Neoplasias) may be useful to confirm the changes we observed in serum miRNA expression and to understand the mechanism by which these miRNAs affect disease progression during the development of cervical cancer. Second, in China, the consciousness of women for cervical cancer screening is relatively weak. Cervical cancer patients are usually diagnosed by pathological results without HPV screening. Thus, we could not analyse the association between HPV infection and miRNA expression. Third, although this article identifies and validates 2 new miRNAs related to cervical cancer, their key target genes and biological functions are not yet clear. Further studies are required to determine the roles of these 2 miRNAs in cervical cancer.

In conclusion, our current study has identified 2 novel miRNAs that are under-expressed in the serum of cervical cancer patients compared with healthy controls. One of these miRNAs enabled the distinction between cervical cancer patients from healthy controls, indicating that it may be a cervical cancer-associated serum biomarker. Our findings are expected to provide clinical references for the mechanistic research into the development of cervical cancer.

## Patients and Methods

### Patients and healthy control subjects

A total of 133 cervical cancer patients and 106 age-matched healthy controls were enrolled in this study from March 2012 to May 2013 at the Chinese PLA General Hospital. All patients were clinically and pathologically diagnosed with cervical cancer. A total of 21 cervical cancer patients and 21 age-matched healthy volunteers were recruited as the training set for constructing serum pools for initial Solexa sequencing (Supplementary table S2). The validation set included 112 cervical cancer patients, 85 healthy volunteers and 55 subjects with other cancers (10 colorectal cancer patients, 16 ovarian cancer patients, 10 breast cancer patients, 7 gastric cancer patients and 12 endometrial cancer patients). All of the healthy controls that were enrolled in the study were women with normal cervixes who had medical examinations in our hospital. We excluded women with recent diagnosis of other malignant tumors, autoimmune disease, pregnancy or chronic wasting disease.

All subjects provided informed consent to participate in the study. This study was carried out in accordance with the approved guidelines by the Hospital Ethics Committee.

### RNA isolation

Blood samples (3 mL) were obtained from the elbow vein from fasting subjects without anticoagulant. After centrifugation at 3,500 g for 7 minutes, the supernatant was retained in cryopreservation tubes and stored at −80°C until use. For Solexa sequencing, each subject provided 900 μL of serum, and then the C1, C2, C3 H1, H2 and H3 groups were pooled. For qRT-PCR, each subject provided 300 μL of serum. Serum total RNA was extracted by mirVana PARIS kit (Ambition, life technologies, Carlsbad, CA, USA), according to the manufacturer's instructions.

### Solexa sequencing

The Solexa sequencing procedure was performed on the above six serum pools (C1, C2, C3, H1, H2 and H3). Among these, C1, C2 and C3 were selected as repeat controls for cervical cancer patients, whereas H1, H2 and H3 were selected as repeat controls for healthy controls. The procedure of Solexa sequencing was carried out as previously described^30^. First, we obtained the desired size ranges of small RNA (under 30 bases) using PAGE purification. We then ligated synthetic oligonucleotide adapters to the 3′ and 5′ ends to enable RT/PCR amplification for 17 cycles. Subsequently, we isolated the products, which were approximately 90 bp (small RNA + adaptors), from agarose gels. Finally, the purified DNA was processed for cluster generation and sequencing analysis using the Illumina Genome Analyzer (Illumina, San Diego, CA, USA) in accordance with the manufacturer's instructions. All procedures were performed at the Beijing Genomics Institute (BGI).

### Computational analysis

After removal of the adaptors, low quality tags and contaminants, clean reads were used for bioinformatic analysis. The small RNA tags were mapped to the human genome using Short Oligonucleotide Analysis Package (SOAP)^33^ to analyze their expression and distribution in the genome. Then, we screened against Rfam 10.1 and GenBank database to remove fragments of rRNA, tRNA, snRNA and snoRNA. After eliminating repeat-associated sRNA and degradation fragments of mRNA and identifying conserved miRNAs, the remained reads which did not match the above databases were predicted using MIREAP (http://sourceforge.net/projects/mireap/). All computational analysis was made by the BGI.

### Novel miRNAs prediction

MIREAP is a computational tool that is specially designed to identify genuine miRNAs from deeply sequenced small RNA libraries^32^. Analyzed sRNAs are only considered candidate miRNA genes if their stem-loop hairpins fulfill the following three criteria: 1) Mature miRNAs are present in one arm of the hairpin precursors, which lack large internal loops or bulges. 2) The secondary structures of the hairpins are stable, with the free energy of hybridization lower than −20 kcal/mol. 3) The hairpins are located in intragenic regions or introns.

To compare the expressions of novel miRNAs between the cervical cancer group and healthy control group, the expressions of miRNA in the two samples were first normalized according to the normalization formula. Normalized expression = Actual miRNA count/Total count of clean reads*1000000. If one miRNA has no read, the normalized read count of this miRNA was set at 0.01^34^; if the normalized expression level of an miRNA was <1 in both the cervical cancer group and in the healthy control group, this miRNA was excluded from subsequent statistical analysis.

All remaining candidates were searched against miRBase 20.0 to rule out known miRNAs. We chose miRNAs which were detected twice in three replicates as our novel miRNAs candidates and used triplet-SVM methods to rule out pseudo-pre-miRNAs. Triplet SVM is a program developed to integrate the triplet element features of a set of real miRNA precursors and a set of pseudo miRNA hairpins^35^. Triplet SVM is used for predicting whether a query sequence with hairpin structure is a real miRNA precursor or not. After analysis by Triplet SVM to remove pseudo-pre-miRNAs, the remaining miRNAs were further validated by qRT-PCR.

### Quantitative RT-PCR

miRNA first-strand cDNA synthesis kits and miRcute miRNA qPCR detection kits (SYBR) (Tiangen Biotech) were used for qRT-PCR validation of the novel predicted miRNAs. All procedures were performed according to the manufactures' instructions. In the miRcute miRNA qPCR detection procedure, the forward primers were synthesized by Tiangen Biotech Company according to different miRNAs, while the reverse primer was same for all the miRNAs. PCR was performed on an ABI PRISM 7300 detection system, and all PCR reactions were performed in duplicate. U6 snRNA was used as an internal control.

### miRNA-gene-network and miRNA-GO-network construction

A miRNA-gene-network and a miRNA-GO-network for the two miRNAs were constructed by Genminix Informatics (Shanghai, China)^36^. Briefly, through target genes function (Gene Ontology, GO) and Pathway-Significant Analysis for the two putative miRNAs, we obtained intersection genes significantly involving GO and the Pathway at the same time. Based on the interactions between the intersection genes and the two putative miRNAs in the Sanger miRNA database, a miRNA-Gene-Network was constructed. The miRNA-GO-network was built according to the relationship of significant GOs and genes and the relationships among miRNA and genes.

### Statistical analysis

The expression levels of miRNAs were calculated by 2^−ΔΔCt^ method as follows: ΔΔCt = (Ct_miRNA_ − Ct_U6snRNA_) _patients_ − (Ct_miRNA_ − Ct_U6snRNA_) _controls_. After testing for normality with Kolmogorov-Smirov and two-sample t tests, data were presented as the median (interquartile range) or mean values and were compared using Mann-Whitney U test or Student *t* test between groups. *P* values of ≤0.05 were considered statistically significant. Receiver Operating Characteristic (ROC) curves were made to determine the diagnostic value. Statistical analyses were performed with SPSS software (version 19.0, IBM, USA).

## Author Contributions

L.J., H.T. and P.Z. wrote the main manuscript text. G.G. was involved in detecting SCC of the serum samples. Z.W. collected the serum samples and data. X.W. and Z.D. gave advice on experiments. Y.T. was involved in designing the study and modifying the manuscript. All authors reviewed the manuscript.

## Supplementary Material

Supplementary Information supplymental files

## Figures and Tables

**Table 1:** Read abundances of small RNAs in C1, C2, C3, H1, H2 and H3 libraries

	Read abundances of total small RNAs					
Category	C1	C2	C3	H1	H2	H3
**Total reads**	13191837	17201872	11517031	14530924	9044505	12042843
**Clean reads**	12655313	14664409	10730467	14039577	8516261	11321127
	96.23%	85.49%	93.52%	96.84%	94.36%	94.20%
**Unique sRNAs**	40566	82548	106967	39455	26117	26126
**exon_antisense**	1154	863	128	609	1429	1532
**exon_sense**	1811	2838	259	1607	2704	4668
**intron_antisense**	4478	5037	689	1997	5991	8048
**intron_sense**	5519	5163	939	2738	8544	11680
**miRNA**	3510595	6458542	731	7478939	4204224	5994620
**rRNA**	55449	255219	15013	136111	53970	102897
**Repeat**	20345	19040	3736	8511	24645	35847
**scRNA**	1940	4393	334	1680	6295	7935
**snRNA**	2589	3819	422	4951	8739	13710
**snoRNA**	202	478	114	358	180	303
**tRNA**	11172	27983	3469	21598	15768	19515
**Unann**	9040059	7881033	81129	6380477	4183759	5120372
	71.43%	53.74%	39.97%	45.45%	49.13%	45.23%

**Table 2:** The information for the 2 predicted novel miRNAs

	miRNA sequences	MFE, k/mol	Length, nt	Primer
**PmiR-1**	CCATGTGTCTGGGCTGGGAAAC	−28.4	22	CCA TGT GTC TGG GCT GGG AAA CAA
**PmiR-2**	TATTGAAAGGCTCCTGGGGAC	−27.8	21	GGT ATT GAA AGG CTC CTG GGG ACA
