# Supplementary material for: Identification and characterization of novel serum microRNA candidates from deep sequencing in cervical cancer patients
Source: Sci Rep. 2014 Sep 3;4:6277. doi: 10.1038/srep06277 (PMC4152751; doi:10.1038/srep06277)
Supplement: Supplementary Information — supplymental files [file srep06277-s1.pdf]

Identification and characterization of novel serum microRNA candidates from deep sequencing in cervical cancer patients

Li Juan[#,1], Hong-li Tong[#,1], Pengjun Zhang[#,1], Guanghong Guo[1], Zi Wang[1], Xinyu Wen[1], Zhennan Dong[1], Ya-ping Tian[*]

1. Department of Clinical Biochemistry, State Key Laboratory of Kidney Disease, Chinese PLA General Hospital, Beijing, China, 100853

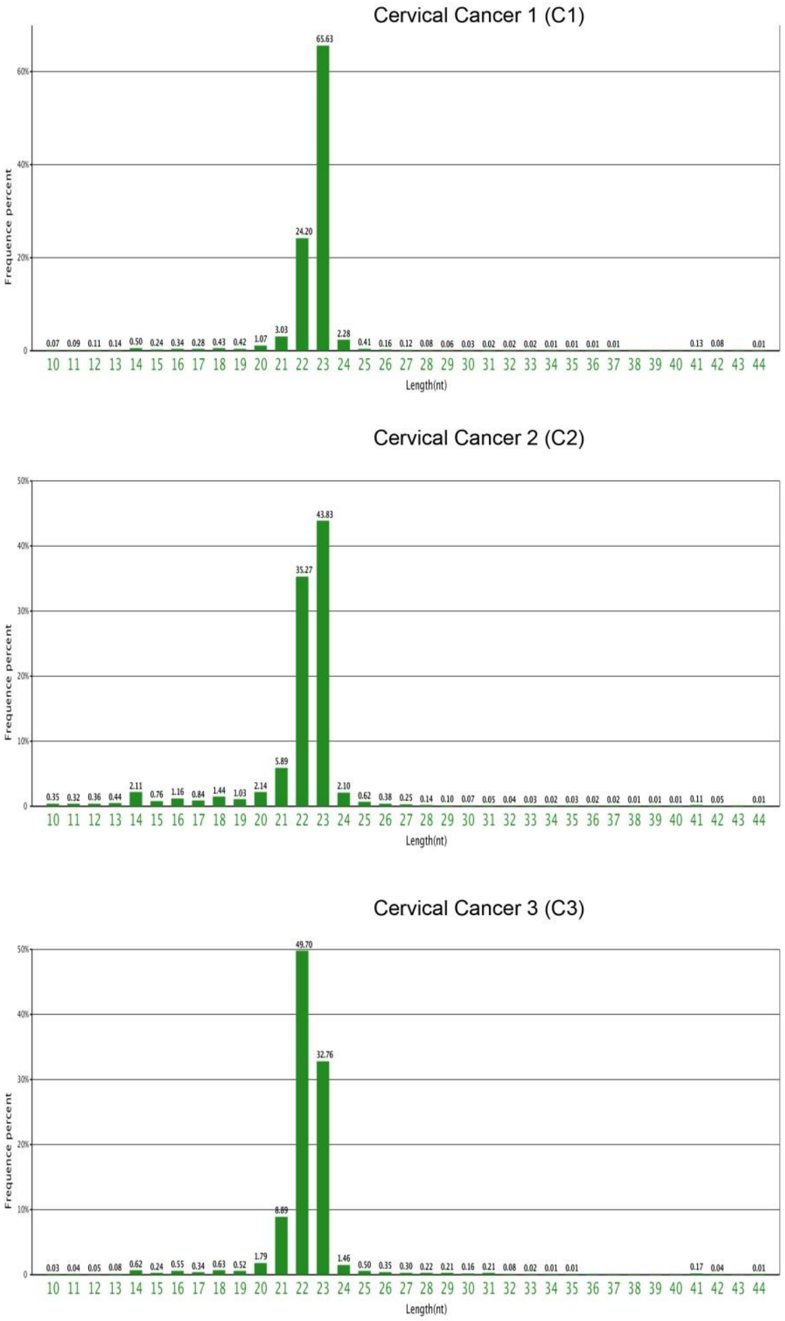

Figure 1. Length distribution for 3 serum pools of cervical cancer patients

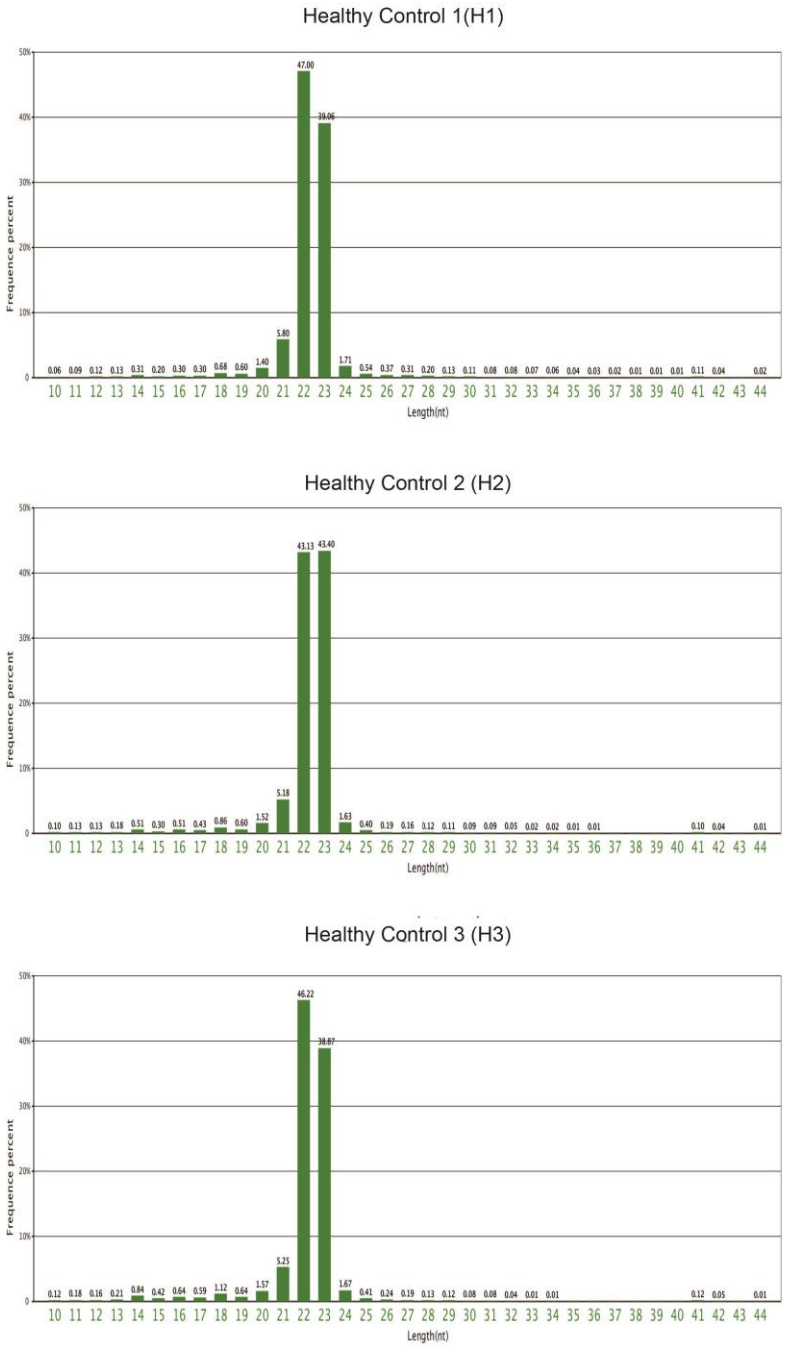

Figure 2. Length distributions for 3 serum pools of healthy controls

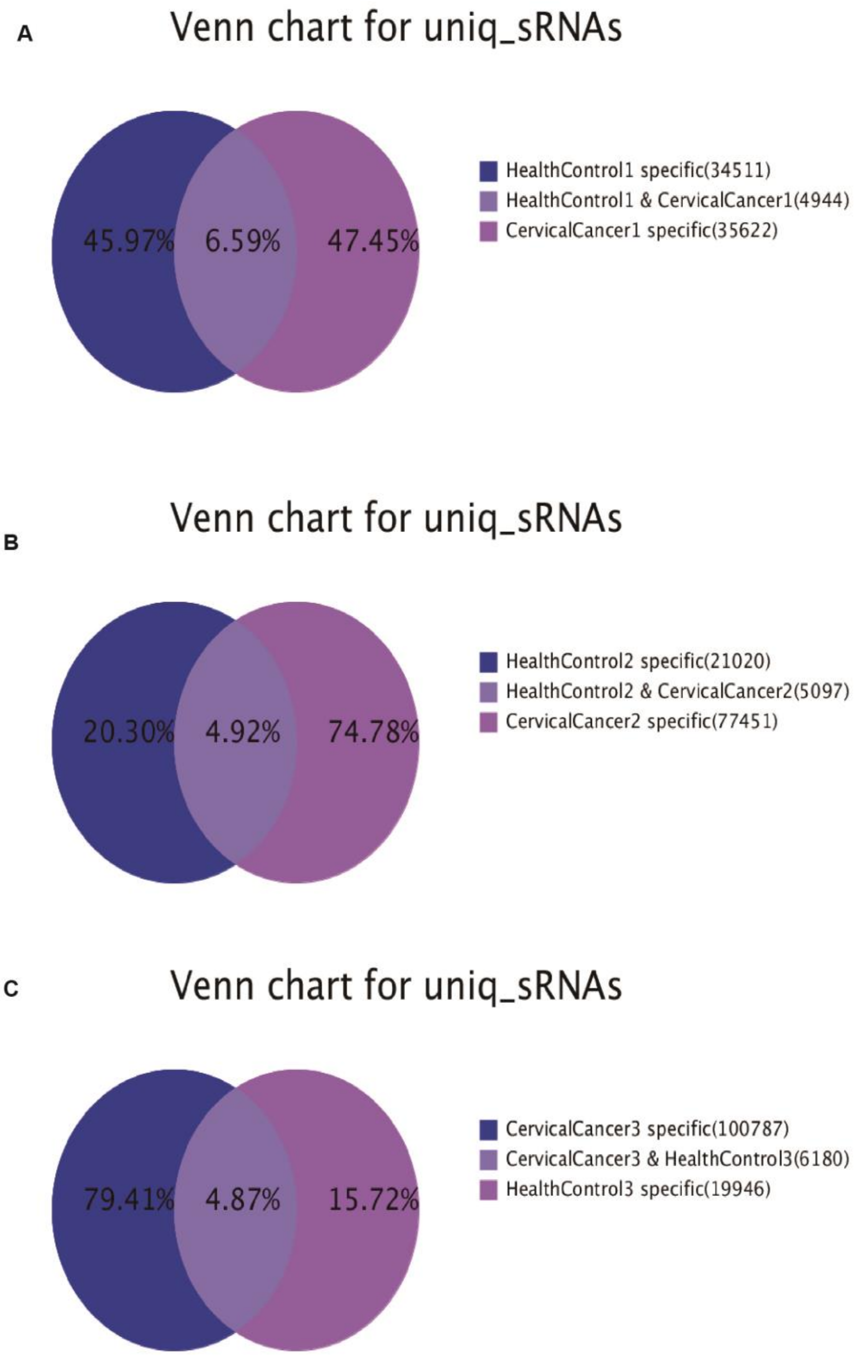

Figure 3. Common and specific of unique small RNA sequence between cervical cancer patients (N=9, each 3 replicate) and healthy controls (N=9, each 3 replicate) detected by Solexa sequencing

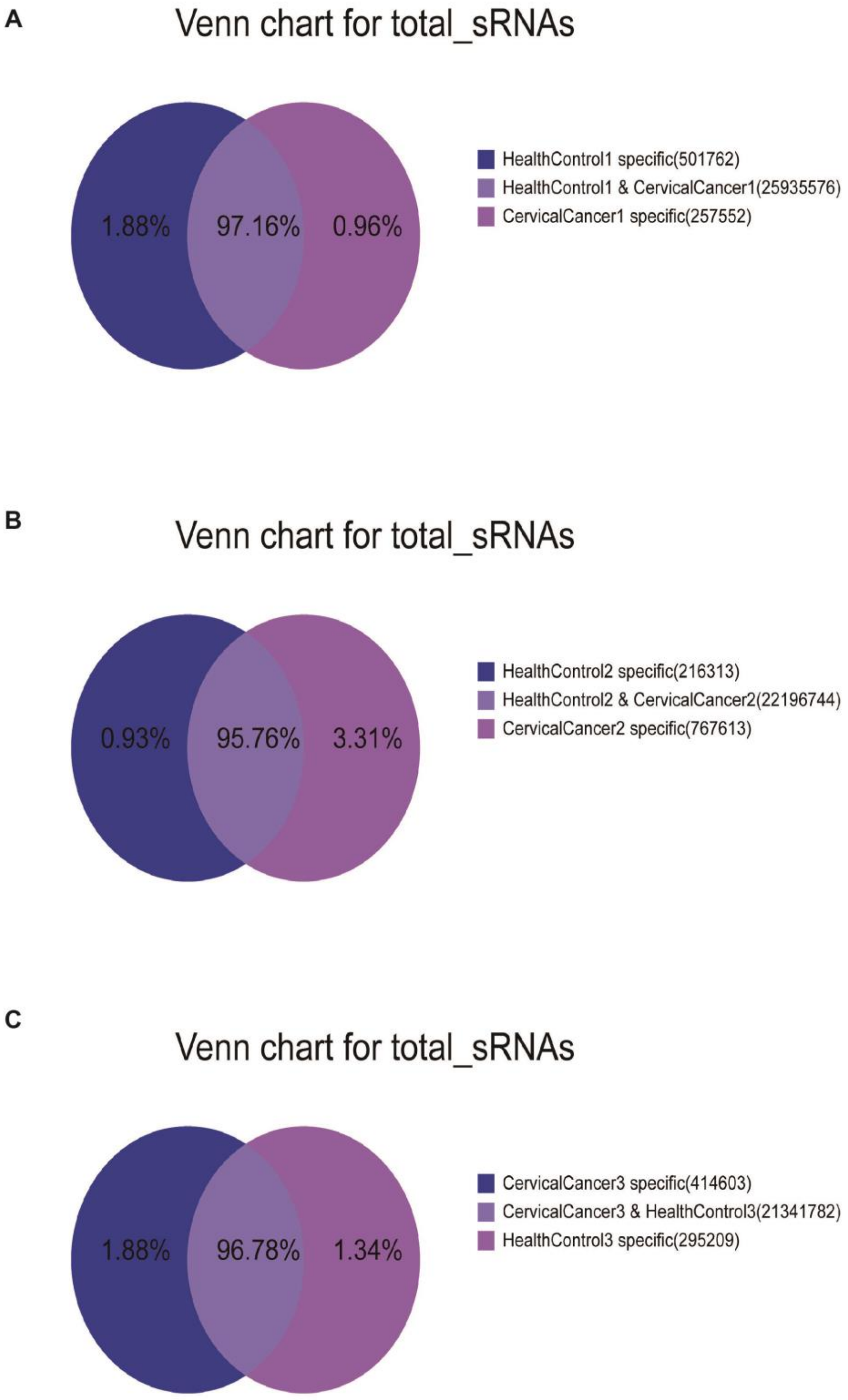

Figure 4. Common and specific of total small RNA sequence between cervical cancer patients (N=9, each 3 replicate) and healthy controls (N=9, each 3 replicate) detected by Solexa sequencing

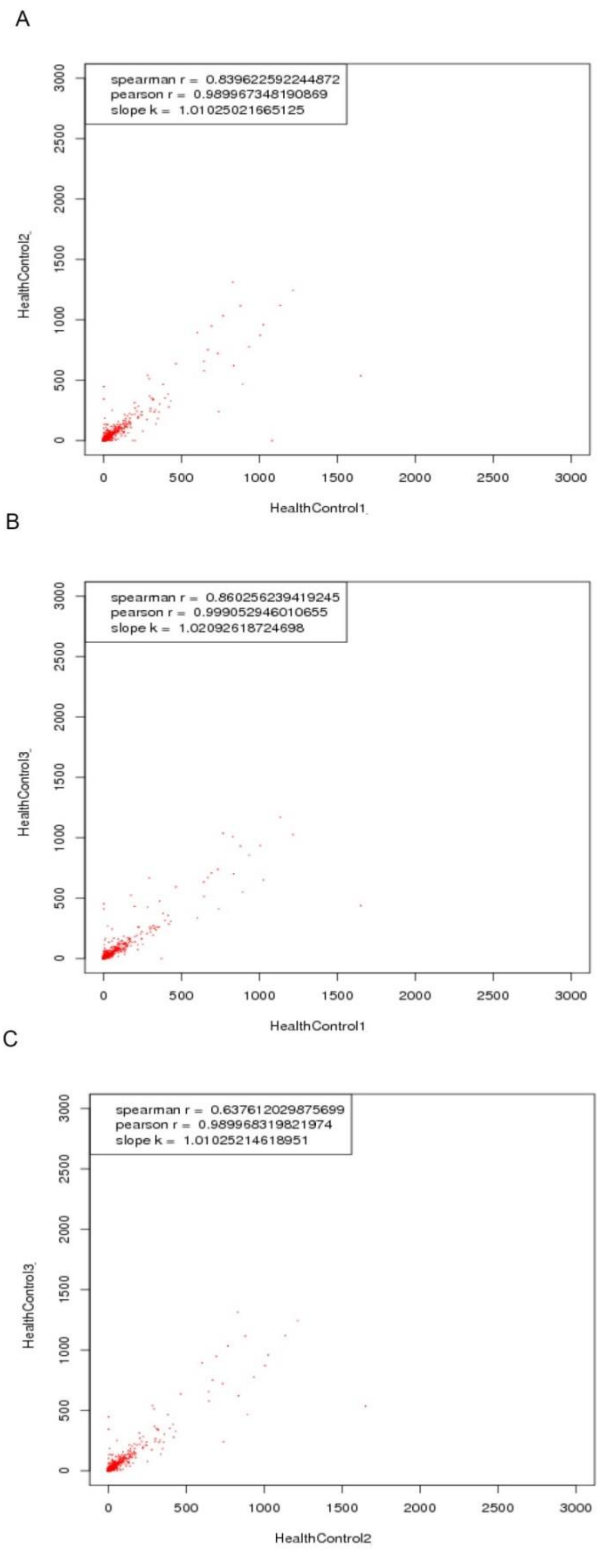

Figure 5. Repeat analysis of the three replicate of serum pools of healthy control group by Solexa sequencing (A) Correlation analysis between serum pools of healthy control group (H1) and group2 (H2); (B) Correlation analysis between serum pools of

healthy control group (H1) and group3 (H3); (C) Correlation analysis between serum pools of healthy control group 2 (H2) and group 3 (H3)

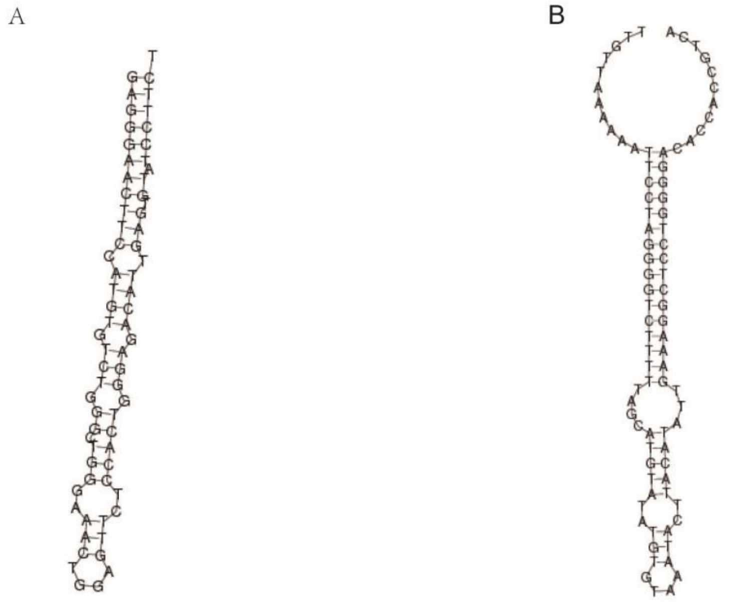

Figure 6. The secondary structure of the 2 novel miRNAs

A, PmiR-1; B, PmiR-2

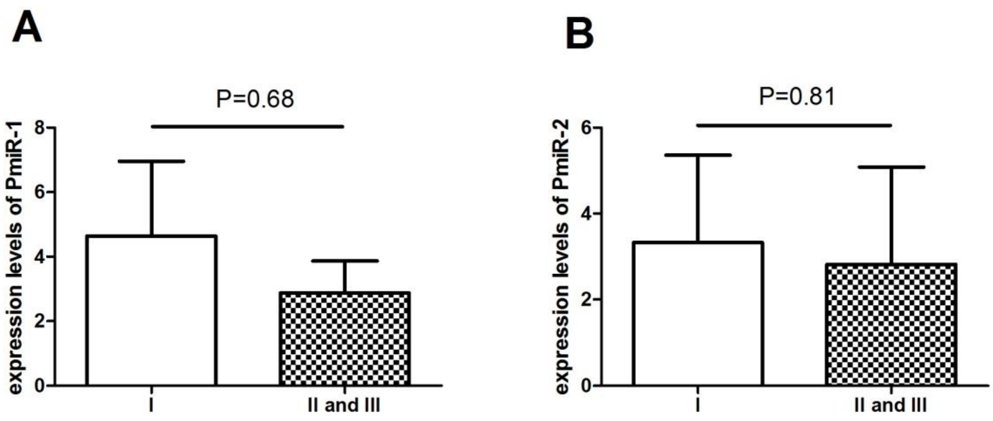

Figure 7. Comparison of the 2 novel miRNAs expression levels between cervical cancer patients with stage I (N=76) and with stage II and III (N=36)

A, PmiR-1; B, PmiR-2

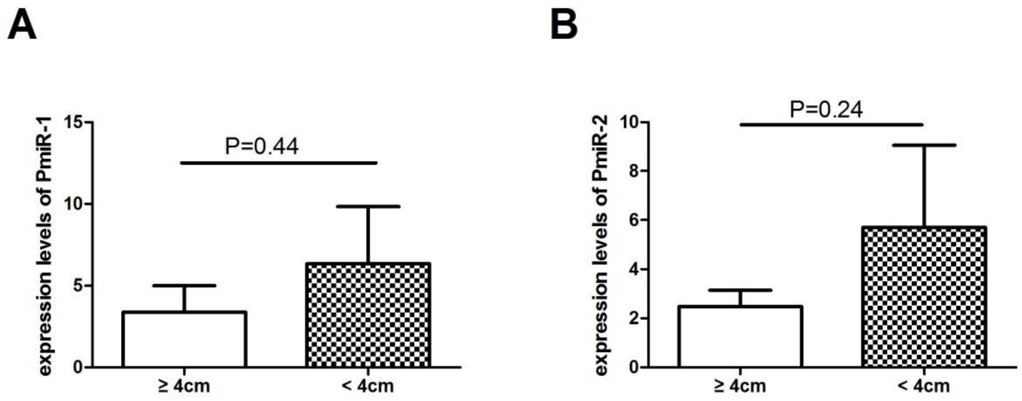

Figure 8. Comparison of the 2 novel miRNAs expression levels between cervical cancer patients with tumor size ≥4 cm (N=20) and tumor size <4 cm (N=59)

A, PmiR-1; B, PmiR-2

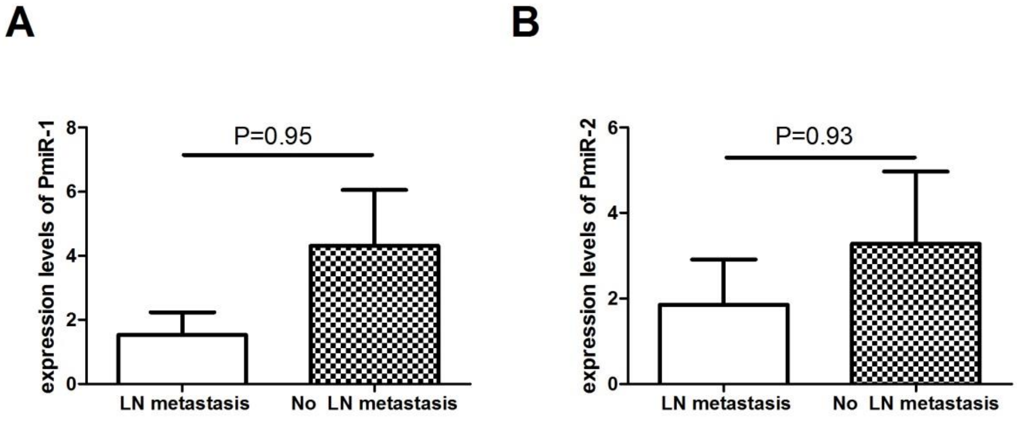

Figure 9. Comparison of the 2novel miRNAs expression levels between cervical cancer patients with positive lymph node metastasis (N=9) and negative lymph node metastasis (N=103)

A, PmiR-1; B, PmiR-2

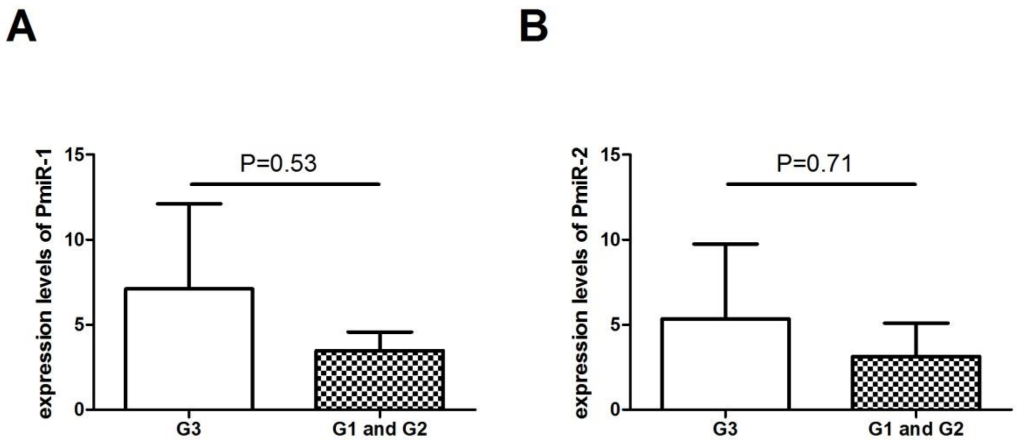

Figure 10. Comparison of the 2 novel miRNAs expression levels between cervical cancer patients with tumor grade G3 (N=9) and tumor grade G1 and G2 (N=103)

A, PmiR-1; B, PmiR-2

Table 1. Clinical data for 112 cervical cancer patients and 82 healthy controls in validation set

	Cervical cancer	Healthy control	P Value
**Female,N**	112	85	
**Age**	47.86±9.88	46.16±10.11	0.172
**U6**	31.19(30.09,32.85)	31.92(30.45,33.30)	0.081
**Stype**			
	Squamous cell carcinoma of the cervix, n=108		
	Adenocarcinoma of the cervix, N=4		
**Clinical stage**			
	Stage I, N=76		
	Stage II, N=34		
	Stage III, N=2		
**Tumor size**			
	≥4cm, N=20		

	<4cm,N=59
	No description, N=33
**Patholgical grading**	
	G1, N=3
	G2, N=39
	G3, N=35
	No description, N=35
**LN metastasis**	
	Positive, N=9
	Negative, N=103

Table 2. Serum miRNAs differentially expressed in cervical cancer cases compared to healthy controls.

	Healthy Control	Cervical Cancer	Fold Change	P Value
**PmiR-1**	20.45 (9.17,68.24)	-4.34 (-5.89, -2.61)	4.08	p<0.0001
**PmiR-2**	3.53 (1.48,11.09)	0.21 (-1.86, 1.64)	3.17	p<0.0001

Table 3. The AUC, Youden index, sensitivity, specificity and 95% CI of biomarkers when diagnosed cervical diseases patients (N=112) from healthy controls (N=85)

Biomarkers	AUC	Youden Index	Sensitivity	Specificity	95%CI
PmiR-1	0.921	0.739	0.857	0.882	(0.883, 0.959)
PmiR-2	0.827	0.586	0.821	0.765	(0.767, 0.887)
SCC	0.690	0.407	0.455	0.952	(0.605,0.776)

Compared to healthy control group, $^{a}p<0.05$
